# Addressing the deficiencies in the evidence-base for primary practice in regional Australia - sentinel practices data sourcing (SPDS) project: a pilot study

**DOI:** 10.1186/1471-2296-14-109

**Published:** 2013-08-01

**Authors:** Abhijeet Ghosh, Karen E Charlton, Lisa Girdo, Marijka J Batterham, Keith McDonald

**Affiliations:** 1 Grand Pacific Health Ltd. trading as Illawarra-Shoalhaven Medicare Local (ISML), Wollongong, Australia; 2 School of Health Sciences, Faculty of Health & Behavioural Sciences, University of Wollongong (UOW), Wollongong, Australia; 3 Statistical Consulting Service, School of Mathematics and Applied Statistics, University of Wollongong (UOW), Wollongong, Australia

**Keywords:** Sentinel sites, Surveillance, Primary care, General practice, Morbidity data

## Abstract

**Background:**

Chronic disease risk on a population level can be quantified through health surveys, either continuous or periodic. To date, information gathered from primary care interactions, using sentinel sites, has not been investigated as a potentially valuable surveillance system in Australia.

**Methods:**

A pilot study was conducted in a single General Practice in a regional area of New South Wales, Australia to assess the feasibility of accessing data obtained through a computerised chronic disease management program that has been designed for desktop application (Pen Computer Systems (PCS) Clinical Audit Tool: ™ PCS CAT). Collated patient data included information on chronic disease management and prevention, prevalence of overweight and obesity, mental health indicators, medication profiling and home medicine reviews, as well as uptake of preventive health services (immunisation and cervical cancer screening).

**Results:**

Higher than national average estimates were found for the age-adjusted prevalence of chronic diseases such as hypertension (14.3% for sample vs 10.4%, nationally), anxiety disorders (4.4% vs 3.8%) and obesity/overweight (67.1 vs 63.4%). Preventive health assessment items were undersubscribed, ranging from 6–20% in eligible patients.

**Conclusions:**

This pilot study has demonstrated that the scope of data collected by patient visits to their General Practitioners, facilitated through the Medicare-funded primary health care system in Australia, offers a feasible opportunity for monitoring of chronic disease prevalence and its associated risk factors. The inclusion of a larger number of sentinel sites that are generalizable to the population being served would provide an accurate and region-specific system for the purposes of population health planning at the primary care level in order to improve the overall health of the community.

## Background

In Australia, under the 2011 Australian Government’s National Health Reform Agreement there has been a recent move for primary care services to be reorganized from former Divisions of General Practice into Medicare Locals throughout the country. These are not-for-profit organisations funded by the Australian Government Department of Health and Ageing (DoHA), that offer a diverse range of primary health care services, through service brokerage and direct delivery and purchasing to improve the health outcomes of the community. The Australian Government also indicates that a Medicare Local is a regional body that undertakes planning to identify the needs of a population and works with local health professionals to provide more integrated care. A more localized surveillance system may improve the understanding of health care needs of local communities, resulting in a more targeted and coordinated approach to meeting these needs through delivery of a range of health, allied health and other services.

In addition, an important part of the National Health Reform was the development of General Practice (GP) Super Clinics [1], aimed at improving access to quality primary healthcare and taking the pressure off the hospital system. These Super Clinics include a greater focus on health promotion and illness prevention, with better coordination between GPs and allied health services, community health and other state and territory funded services. Entities, including Medicare Locals, were invited to apply for Federal government funding to establish and develop the GP Super Clinics at various sites across Australia. Amongst others, the Illawarra Shoalhaven Medicare Local (ISML), located in a regional area of New South Wales, obtained funding to establish the first GP Super Clinic in the region. The facility opened for patients on 6^th^ July 2011, trading as Shell Cove Family Health (SCFH) [2].

**Figure 1:**
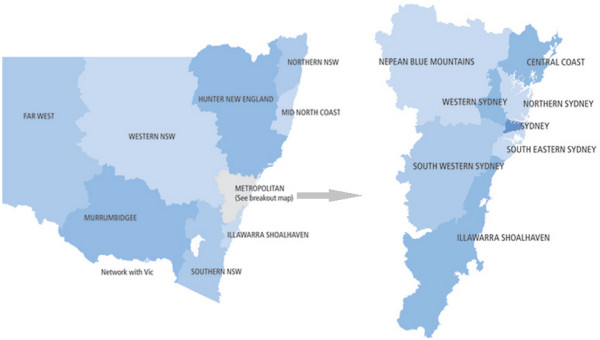
**Local health district boundaries of New South Wales, Australia.** Source: [6]

**Figure 2:**
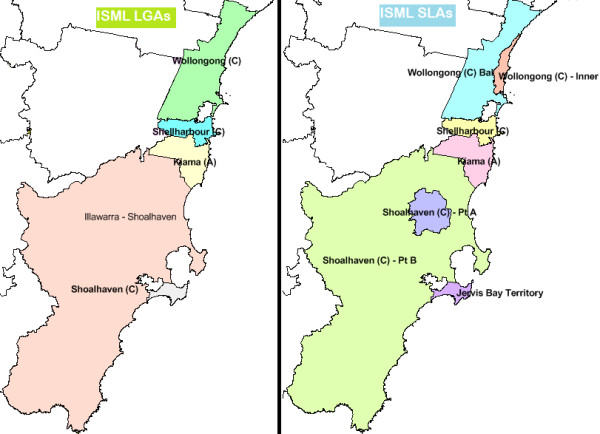
**Illawarra-Shoalhaven Medicare Local: Constituent Local Government Areas (LGAs) and Statistical Local Areas (SLAs).** Source: Adapted from [5]

Valid data on morbidity, at the regional level, is essential for the purposes of primary healthcare services planning, that are specifically tailored to the needs, demands and requirements of the local population. Nationally representative data is available through the National Health Surveys (NHS) conducted by the Australian Bureau of Statistics (ABS) [3]; and regionally through the annual New South Wales Population Health Surveys [4]. However, extrapolations of these data to smaller geographical areas such as Local Government Areas (LGAs) and/or small area geographic regions within LGAs like suburbs and Statistical Local Areas (SLAs) is limited. The state of New South Wales (NSW) has 15 Local Health Districts (LHDs) and 17 Medicare Locals (MLs). While the LHDs run the public hospitals across the nation and are responsible for acute, sub-acute and palliative care services, the MLs are responsible for primary healthcare planning and delivery for their constituent LGAs. Some of the densely populated LGAs are further sub-divided into constituent SLAs. For illustration, a map of the NSW Local Health Districts and a breakout map of the Metropolitan LHDs is shown in Figure 1. Within the Illawarra Shoalhaven region, distribution of the constituent LGAs and SLAs within the ISML and the Illawarra Shoalhaven Local Health District (ISLHD) catchment areas is shown in Figure 2[5].

The Public Health Information Development Unit (PHIDU) at the University of Adelaide compiles and provides synthetic predictions of the prevalence of chronic diseases and associated risk factors at LGA and SLA levels but these are produced using modelling techniques based on the survey data collected in the 2007–08 ABS National Health Survey (NHS) and known characteristics of smaller areas at that time attained from the ABS Census. A synthetic prediction is provided according to SLA, and is argued as being the likely value for a ‘typical’ area with similar characteristics. These techniques provide reasonable estimates of disease burden, but are limited in terms of planning health care services for niche groups within further smaller regions represented by SLAs or suburbs.

Moreover, because the NHS sample excludes the most remote areas of Australia, synthetic predictions cannot be generated for smaller geographic local areas with relatively high proportions of their population in remote areas. Thus, while national representation of the NHS is excellent, regional and smaller area figures devised from the survey data are estimations at best and have a significant likelihood of being inaccurate for regions with diverse and disparately distributed populations.

Epidemiological indicators are also measured in the ongoing NSW Population Health Surveys that has been conducted by the NSW Ministry of Health since 1997 for adults and since 2001 for children. However they too are reported at a state-wide level and further broken down to Local Health District levels only. Measures for SLAs and LGAs are neither available, nor is it statistically accurate, to be extrapolated from state-based prevalence figures. The sampling frame for the NSW Population Health Surveys is approximately 1,000 persons in each of the health administrative areas (Local Health Districts). Hence for every indicator a respondent sample size of 8000–16000 is obtained for the entire state of NSW.

Furthermore, all the estimates drawn from national and state level surveys are based on self-reported health and disease status data as provided by respondents of these surveys. The accuracy of self-reported data especially in terms of health services planning and mapping of regional disease epidemiology has always been questioned by health planners [7]. On the other hand disease diagnosis as noted in clinical softwares within hospitals and primary health care settings is clinically diagnosed and is supported by clinical diagnostic testing, examination and medical symptomatic evidences. Hence health data that is validated by the clinical judgement of medical practitioners offers a significantly higher level of accuracy of disease prevalence and is likely to be far more accurate than self-reported health information. Collation and analysis of clinical data thus provides a more evidence based platform for chronic disease surveillance for primary health care providers and for researchers to study regional epidemiology.

In much the same way that registries based in secondary care provide surveillance for cancers and other diagnosed diseases, there are examples of surveillance systems for chronic diseases located within primary care facilities from various parts of the world [8-15]. Data collected routinely from sentinel sites (general practices) allows generalizable data to be collected from representative samples instead of the entire population. However, the success of sentinel site surveillance systems relies on motivation of participating practitioners, a well-functioning clinical practice database that allows for rapid data extraction, and timeous data collation and reporting.

The current pilot project aimed to assess the feasibility of implementing a sentinel site surveillance system within the newly formed GP Super Clinic, namely Shell Cove Family Health (SCFH), located in the Shellharbour LGA of the Illawarra-Shoalhaven catchment of New South Wales, Australia.

## Methods

The Pen Computer Systems (PCS) Clinical Audit Tool™ (CAT) is a program that has been designed to collect and collate population-level health data for the purpose of chronic disease management. The program can be installed onto compatible desktop computer systems within General Practice and interfaces with the practice’s clinical software. It thus helps to simplify the review of clinical and practice management databases through analysis of practice data to identify chronic disease-related episodes of care, and associated risk factor prevalence. All practice clinical softwares utilise one of the several nationally validated health coding and medical classification systems, such as SNOMED-CT, DOCLE, PYEFINCH and ICPC2+. These are commonly known as medical vocabularies. Clinicians enter patient data using their practice software, according to these medical vocabularies, thus facilitating assessment of existing disease and health risk factor prevalence that is recorded during patient encounters at the practice. Higher level data analysis that is made possible through the program includes:

•collection and aggregation of National Performance Indicators by Divisions of General Practice (now Medicare Locals);

•collection and aggregation of data for research by Health Communication Network Pty Ltd through their General Practice Research Network initiative;

•collection and aggregation of research data required by governments and other agencies which has passed through an ethics approval process such as that offered by the Royal Australian College of General Practitioners (RACGP); and

•collection and aggregation of practice data by Pharmaceutical companies.

In all cases, judgments about the secondary use of practice data for these types of initiatives are made by General Practitioners. The PCS CAT has an automated de-identifying feature ensures privacy of patient information, allowing data to be extracted for research purposes.

The selected pilot site for the project was the newly formed GP Super Clinic - Shell Cove Family Health (SCFH) located in the Shellharbour LGA of the Illawarra-Shoalhaven catchment of New South Wales, Australia. The practice clinical software was *Best Practice* which uses the PYEFINCH medical coding vocabulary. Practice staff were informed about the aims and objectives of the study assisted the researchers in undertaking the data cleansing process on their clinical software system. The data cleansing phase of the study was conducted using the data maintenance utility tool which is available within all GP clinical software. Within *Best Practice* this is called the *Cleanup history* tool. Data cleansing included:

•encouraging all practice staff to use the ‘drop down box functionality’ of *Best Practice* to define all medical diagnoses and other sections of the patient record;

•strictly avoiding free text entries in all sections of the patient record;

•finding all identifiable free text non-coded past medical history items, and either linking them to appropriate coded items or replacing them with the correct coded item; and

•coding all inactive patients as ‘Inactive’. An ‘active patient’ is one who has attended the practice three or more times in the past two years as defined in the RACGP Standards for general practices [16].

While incomplete history-taking by the clinician may under-estimate cases of chronic disease, the data cleansing process which involves the systematic recording and allocation of coded diagnoses allow the practice clinical software to draw on the coded information so as to attain the best possible coverage in terms of accurate case identification. For example, if a patient with Type 2 Diabetes Mellitus was not coded as such in the clinical system using pre-coded options, and Type 2 Diabetes was merely inserted as a free text entry in the patient’s record by the GP, this count of Type 2 Diabetes would be missed, thus introducing bias. The free text entry which has been inserted by the GP as per his clinical judgement, will then be replaced to the coded item of ‘Type 2 Diabetes Mellitus’ during the data cleansing process and hence the case will be illustrated as one of Type 2 Diabetes within the data extract and will be available as such during analysis. Such cleaned and correctly coded information is then easily extractable from the *Best Practice* clinical software using the PCS CAT tool.

A cleaned, de-identified PCS CAT data extract was performed in July 2012 which included all information obtained from patient interactions in the preceding 15 months for all diagnosed pathologies, clinical variables such as anthropometric measures, patient demographic information such as age, sex, geographical location of residence (postcodes) and indigenous status. Extracted data was converted to usable database formats and then analysed using Microsoft Excel (V2007: Microsoft Corporation, Redmond Washington, USA) and IBM SPSS Statistical program (V19.0: 2010, IBM Corporation, New York, USA). The resultant SPSS and Excel databases hence included clinical diagnosis and patient demographic information as entered by GPs of the pilot site.

Basic epidemiological measures in the form of age specific prevalence and total prevalence were then calculated for all individual diagnosed conditions. These prevalence figures were matched and analysed with comparable indicators for same age groups as reported by the Australian Health Survey (AHS) 2011–12 conducted by the Australian Bureau of Statistics (ABS) [17]. The age specific disease prevalence figures obtained from the sample and the estimated national prevalence figures reported by the AHS 2011–12 were then age standardised using the 2011 estimated resident population of Australia [18]. Comparisons across age standardised prevalence were conducted for all major chronic conditions that the SPDS project is targeting for regular surveillance namely, obesity; overweight; diabetes mellitus; hypertension; asthma; mental health disorders such as clinically diagnosed depression and anxiety disorder; Coronary Heart Disease; Stroke; and Chronic Bone Diseases such as Osteoarthritis and Osteoporosis. Both Microsoft Excel (V2007: Microsoft Corporation, Redmond Washington, USA) and the PCS CAT tool (v.3.1: pencs.com.au) were used for graphical illustration of demographic data and age specific disease prevalence.

Other patient interactions that were recorded and analysed for relevant age specific cohorts were the uptake of preventive health checks as endorsed by the Australian Governments’ Medicare Benefit Scheme (MBS) Items [19] under the group: Health Assessments. Using the MBS schedule of January 2012 [19] and the MBS items claiming patterns of the practice for the actual health assessments undertaken, a potential revenue estimation matrix was created using Microsoft Excel (V2007: Microsoft Corporation, Redmond Washington, USA) and estimations of potential revenue lost were calculated in order to illustrate the added benefits of improving preventive health item uptake for the practice.

The study was performed with the approval of the Human Research Ethics Committee (Health and Medical) of the University of Wollongong (HE 12/447).

## Results

The number of patients that had visited the General Practice within the previous 15 months (30^th^ April 2011 to 31^st^ July 2012) was 3623 (1574 men; 2041 women; 8 no gender identified).

**Figure 3:**
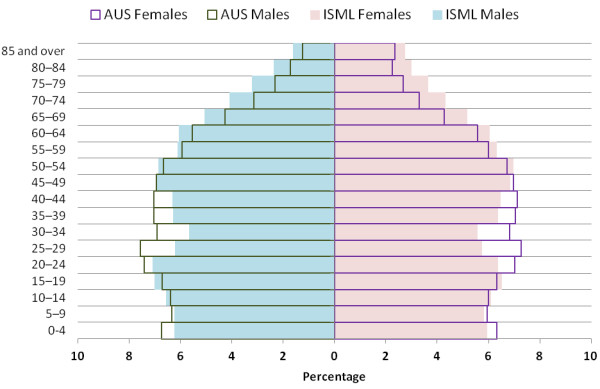
Population pyramids of the Illawarra-Shoalhaven population compared to national Australian population.

**Figure 4:**
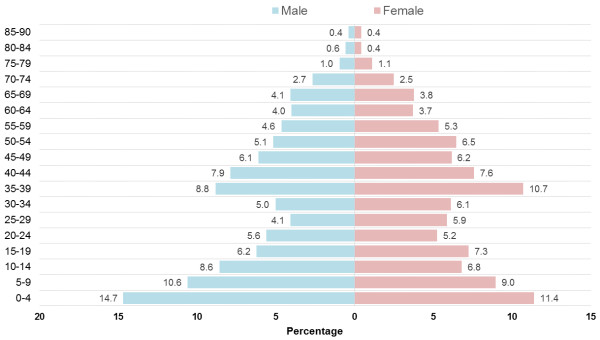
Population pyramid of the sample included in the pilot General Practice.

Median age for the sample was = 32 (IQR = 11–48) years. Children aged 0–4 years comprised the largest age group at 12.9% of the total sample, followed by the 35–39 year age group (9.9%), and 5–9 year old children (9.7%). Older adults aged 65 years and above comprised 8.5% of the sample. The age distribution of the population of the Illawarra Shoalhaven Medicare Local (ISML) coverage region is compared against that of the total Australian population in Figure 3, and the population pyramid of the sample is shown in Figure 4. The proportion of the local population who consulted the pilot practice during the study period is shown in Table 1. Proportions are based on the residential postcodes of the persons within the sample. A significant majority of the sample (72.3%) resided within the practice location postcode area and most of the remainder came from surrounding postcodes within the Shellharbour, Kiama and Wollongong LGAs.

**Table 1:** **Proportion of local population that consulted the general practice during the 15 months (30**^**th**^**April 2011 to 31**^**st**^**July 2012)**

**Postcodes of persons in the sample**	**No. of patients from the postcode within the sample**	**Proportion of the sample from the postcode (%)**	**Usual resident population of the postcode**	**Proportion of the local population included in the sample (%)**
2529^	2,618	72.3	22,309	11.7
2533*	230	6.35	14,938	1.5
2528^	205	5.66	23,283	0.9
2527^	140	3.86	20,614	0.7
2534*	127	3.51	4,991	2.5
2530^#^	44	1.21	29,720	0.1
**Total**^**~**^	**3364**^**~**^	**92.9**^**~**^	**115,855**	**2.9**

^**~**^Postcodes that contributed to less than 1% of the sample are not included in this table.

^Postcode is within Shellharbour LGA.

*Postcode is within Kiama LGA.

^#^Postcode is within Wollongong LGA.

**Figure 5:**
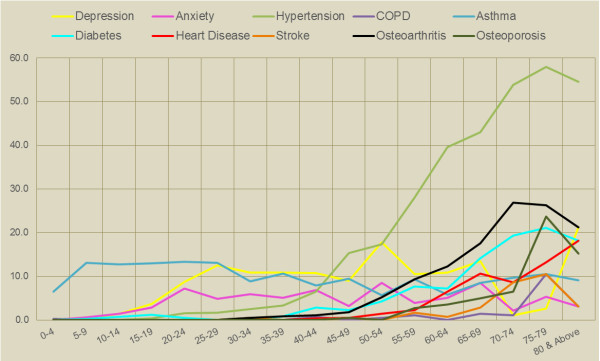
Age specific prevalence of chronic diseases within the sample.

The age specific population and disease counts within the sample and the age standardised prevalence comparisons of the sample and the Australian National estimates are shown in Tables 2 and 3 respectively. An illustration of age specific burden of disease (Figure 5) indicates that asthma and depression prevalence is much higher amongst younger age groups compared to older adults. While the proportion of older aged persons was comparatively smaller than middle aged adults (Figure 4), the proportional share of conditions such as hypertension, osteoarthritis, osteoporosis and diabetes are much higher in older age groups.

**Table 2:** **Age specific population and disease counts within the sample during the 15 months (30**^**th**^**April 2011 to 31**^**st**^**July 2012)**

**Age groups**	**Denominator (n)**	**Depression**	**Anxiety**	**Hypertension**	**COPD**	**Asthma**	**Diabetes**	**Heart disease**	**Stroke**	**Osteoarthritis**	**Osteoporosis**
0-4	465	0	0	0	1	30	0	0	0	0	0
5-9	351	1	2	0	0	46	1	0	0	0	0
10-14	275	4	4	0	0	35	2	0	0	0	0
15-19	247	9	7	1	0	32	3	0	0	0	0
20-24	196	17	14	3	0	26	1	0	0	0	0
25-29	184	23	9	3	0	24	0	0	0	0	0
30-34	204	22	12	5	1	18	0	0	1	1	0
35-39	359	39	18	12	0	38	3	0	0	3	0
40-44	280	30	19	18	2	22	8	1	0	3	0
45-49	222	20	7	34	0	21	5	1	1	4	1
50-54	213	38	18	37	1	12	9	3	0	11	0
55-59	182	19	7	51	2	17	14	4	3	17	5
60-64	139	15	7	55	0	8	10	9	1	17	5
65-69	142	19	12	61	2	12	20	15	4	25	7
70-74	93	1	2	50	1	9	18	8	8	25	6
75-79	38	1	2	22	4	4	8	5	4	10	9
80 & Above	33	7	1	18	1	3	6	6	1	7	5
**Total**	**3623**	**265**	**141**	**370**	**15**	**357**	**108**	**52**	**23**	**123**	**38**

**Table 3:** Age standardised disease prevalence of the sample compared to national averages

**Chronic disease/ conditions (as defined and entered into electronic records by GP)**	**Age standardised disease prevalence of sample (%)**	**Australian National health survey estimate (%) 2011-12**
Depression	8.7	9.7
Anxiety	4.4	3.8
Hypertension	14.3	10.4
COPD	0.7	2.4
Asthma	9.9	10.2
Diabetes	4.2	4.0
Heart disease	2.4	4.9
Stroke	1.0	1.1
Osteoarthritis	5.0	8.4
Osteoporosis	2.0	3.4
Obesity (BMI ≥ 30)*	30.8	28.3
Overweight and or obese (BMI ≥ 25)*	67.1	63.4

*Adults only.

The number of preventive health assessment items, reimbursable by the Medicare Benefits System, that were performed during the recording period are shown in Table 4. While the sub-optimal uptake of preventive health checks indicates a lost opportunity to check on the health and well-being of identified vulnerable cohorts within the practice patient population, it also reflects on the missed revenue earning potentials for the practice. Potential revenue lost quantified using the assumptions drawn from the MBS items claiming patterns of the health assessments undertaken by the practice are illustrated in Table 5.

**Table 4:** **Uptake of MBS group A14 (Health assessments) items during the 15 months (30**^**th**^**April 2011 to 31**^**st**^**July 2012)**

Item categories*	**Eligible population within sample**	**Health assessments undertaken**	**Uptake in eligible patients (%)**
Healthy kids check	172	35	20.3
45-49 year health check	222	13	5.9
75+ health check	71	10	14.1

*Diabetes health assessment has been omitted due to lack of diabetes risk assessment data.

**Table 5:** **Potential revenue lost from uptake of preventive health MBS items during the 15 months (30**^**th**^**April 2011 to 31**^**st**^**July 2012)**

**Item categories**	**701**	**703**	**705**	**707**	**TOTAL**	Conservative assumptions*	**Potential revenue lost**
	**($)**	**($)**	**($)**	**($)**	**($)**		**($)**
Healthy kids check	456.80	10,881.40	5,674.55	4,138.40	21,151.15	*75%*	15,863.36
45-49 year health check	0.00	21,364.70	5,857.60	4,138.40	31,360.70	*25%*	7,840.18
75+ health check	0.00	1,592.40	3,294.90	8,018.15	12,905.45	*75%*	9,679.09
**Total preventive health items**							**33,382.63**

*Realistic assumptions have been employed which accept that 100% patient coverage is neither possible nor feasible. Assumptions were based on the perspectives from the practice’s GPs, Nursing and Management staff. Hence a percentage share of the estimated revenue from Health Assessments have been used to calculate the reportable *Potential revenue lost*.

## Discussion

The Pen Computer Systems (PCS) Clinical Audit Tool™ (PCS CAT) offers the ability to collect and collate population-level health data for the purpose of chronic disease management. Through engagement with primary health services, data extraction may facilitate better planning for preventive as well as curative health services. To date, the desktop computerised system has been adopted by 62 Divisions of General Practice (now merged into lesser or equivalent numbers of Medicare Locals) throughout Australia. Its primary purpose is to provide Divisions the ability to audit their members’ National Performance Indicator (NPI) requirements, for funding allocations through Medicare reimbursements. Additional uses include the measurement of epidemiological indicators at a local and regional level for planning purposes, the uptake of various services by patients, including preventive strategies, and the ability to measure the effectiveness of programs delivered through general practice. This pilot study compared the prevalence of chronic diseases, and related risk factors, such as overweight and obesity and mental health indicators with data collected in recent national and regional surveys. It also assessed the uptake of primary prevention initiatives, including cervical screening, immunisations, medication profiling and home medicine review.

This data obtained from patient interactions provides an accurate and timely picture of the major primary health care needs of patients that access general practitioner services within an identified smaller geographic region within a local government area. For example, it is clear that activities that target weight reduction and prevention of hypertension are required in the catchment area of this General Practice. Compared to estimates obtained from the ABS Australian Health Survey 2011–12, this pilot study identified higher than national figures for the prevalence for clinically diagnosed anxiety, hypertension, and overweight and obesity, but a lower prevalence of asthma, COPD, chronic heart disease, depression, osteoarthritis and osteoporosis. The prevalence of diabetes was similar to Australian national estimates. While these prevalence figures represent only the section of the population that goes to a GP and can thereby be argued to be a biased estimate of regional population disease prevalence; the figures do help primary care initiatives to target vulnerable groups that present to general practices with an identified need to address specific chronic conditions. They hence enable primary health planners to tailor healthcare services to meet local needs of the populations that actively visit general practices to receive primary healthcare services. It is not possible to determine from this single pilot practice data source whether the age-standardized prevalence figures are truly reflective of regional differences, or whether they may be a result of some disease classification and coding anomalies, nor is it possible to hypothesize why such differences may exist. This will be a topic of further larger studies.

The collection of morbidity data in primary care can be based on either (a) episodes of care, whereby patient interactions are recorded, or (b) a limited number of specific disorders that are standardized using case definitions, usually defined according to the nationally recognised disease coding systems. The former provides information on patterns of clinical management, such as the method employed in the English General Practice Research Database Programme [11,20], whereas the latter provides a picture of the burden of disease in the population, as in the Morbidity Sentinel Stations Programme that operates in several European countries [13-15,21,22]. In both these surveillance models, data generated from general practice settings have been shown to be useful [23]. Our pilot study obtained data using both of these models, however broader application of the PCS CAT system for the purpose of surveillance will require further streamlining of the type of data to be extracted. It could be argued that morbidity data should be based on individuals rather than the patient-provider consultations as the latter reflects workloads and case management rather than burden of disease in the epidemiological sense [11].

In Australia, as in most other countries, general practitioners are the first source of referral within the larger health care system, and thereby provide an optimal opportunity for effective monitoring of morbidity data of the population. The internal validity of the surveillance programme will depend on the accuracy of classification of disease at the point of data entry by practitioners, together with a reliable source of denominator data. An example of how inconsistencies in definitions of hypertension can impact on variations in age-adjusted prevalence figures and estimates of hypertension control collected in surveillance systems is described by Crim and colleagues [24]. Different definitions, using data collected in the National Health and Nutrition Examination Surveys (NHANES) (2003/4 onwards) has resulted in varied estimates of prevalence, ranging from 29% to 32%, and levels of hypertension control, from 35% to 64%.

In France, the Public Health Act of August 9, [25] listed 100 health targets for a 5-year period, and is currently under review for updated health targets. Evaluation of the effectiveness of public health programs to meet these targets requires representative data on indicators of infectious and chronic diseases chronic diseases at a regional level. The existence of a national health identification number and the development of a national information system inter-operability framework that has the capability to interchange data between heterogeneous systems, paves the way for implementation of such an epidemiological surveillance system. The Rhône-Alpes regional health platform that began in the 2000s and by August 2011, had 2.6 million patients in its repository, provides a feasibility study for widespread implementation of regional platforms for managing electronic health records [26].

Sharing of electronic health records between care facilities to improve health care coordination has been the object of major investment in many countries. Programs have been launched, and are at various phases in development and implementation in Australia (the national HealthConnect program), New Zealand (national program), the United Kingdom (national program managed within the National Health Service), France (personal medical record or DMP project) and the United States. Projects in these countries are currently in various phases of development and/or implementation with none being fully operational as yet. An added, but still to be explored, major benefit of these interfaced data recognition systems will be epidemiological monitoring and surveillance. In the US, the Centers for Disease Control and Prevention (CDC) set up a National Center for Public Health Informatics in 2005, but reports that the “fragmentation of population health data collection, and data stewardship responsibilities among federal, state and local governments” [27] remains the greatest barrier to a creation of a population health record (popHR) in the United States. The definition of a popHR is “*…aggregated and usually de-identified data. It may be obtained directly from EHRs or created de novo from other electronic health repositories. It is used for public health and other epidemiological purposes, research, health statistics, policy development, and health services management*” [28,29]. We propose that the PCS CAT system provides a feasible method by which to obtain a popHR in the Australian primary care setting.

The PCS CAT system also offers potential to monitor uptake of preventive health activities that are offered through the existing Medicare Benefits Scheme (MBS). Reimbursement is provided to general practitioners for 4 time based MBS health assessment items: 701 (brief), 703 (standard), 705 (long) and 707 (prolonged) [30]. Under these items a medical practitioner is able to undertake a range of health assessments including: Healthy Kids Check (children aged 3 – 5 years, or who are receiving their 4 year old immunisation); a health assessment for people aged 45–49 years who are at risk of developing chronic disease; a type 2 diabetes risk evaluation for people aged 40–49 years with a high risk of developing type 2 diabetes as determined by the Australian Type 2 Diabetes Risk Assessment Tool (AUSDRISK) [31]; and a health assessment for people aged 75 years and older (75 + HA) [30]. Our data has identified a low uptake of these MBS-funded items by eligible patients in the practice, despite a recent focus on preventive health care, as evidenced by significant government investment in the establishment of an Australian National Preventive Health Agency [32]. Australia is one of a number of countries that have recognised the need to re-orient health systems from a treatment model to a preventive health-promoting model. However, our data suggests that within primary care, there is a need to encourage individuals to access these health checks as a starting point for dialogue around healthy lifestyles.

The main limitation of the study relates to validity and generalizability of the prevalence data. This pilot study was undertaken to assess the feasibility of obtaining prevalence data on chronic disease conditions using data that is recorded by practitioners during episodes of care, rather than providing a comprehensive snapshot of the population. Generalizability of data collected from sentinel sites requires representativeness of the general practice clientele to the broader population in that geographical area, which will require statistical techniques to identify practices, both large and small, that are relative to the size of the populations that they serve. This approach will be adopted in the same geographical region as a follow-on from this pilot project.

One of the main benefits of using general practice databases for disease surveillance is the ability to access data from large patient populations across a wide population coverage. However, the data are collected primarily for clinical and routine use, rather than for surveillance or research purposes. Hence, data quality and reliability may be compromised [33] and additional data cleaning is required before being extracted to a usable format, as was performed in the present study. The SPDS study relies on the clinical judgement of GPs for accuracy of disease diagnosis and assumes inclusiveness of GP history-taking, clinical inquiry and data recording skills. The validity of medical terminology and coding systems such as PYEFINCH, DOCLE and ICPC2+ also need further standardisation. With the introduction and implementation of new e-health requirements in Australia in February 2013, the National E-Health Transition Authority (NEHTA) has advised all General Practice clinical software vendors to start working towards mapping their local medical vocabulary against SNOMED-CT, that has been identified by NEHTA as the preferred nationally recognised disease classification or terminology system [34]. In due course, coded General Practice clinical data in Australia may be considered a reasonably valid and reliable source of information for mapping regional disease prevalence and conducting surveillance of chronic conditions. Such data is particularly useful for planning of primary care services to meet the needs of local populations.

A limitation of the proposed sentinel site surveillance system is that the PCS CAT programme is not yet installed in many general practices across Australia. Staff working in the pilot site in the current study received training and assistance to improve the accuracy of the data extracts. Researchers manually trained the practice staff who then undertook data cleaning, which essentially meant ensuring that the information in an individual patient’s medical record was stored in a way that allowed the record to be searched, thus improving the quality of information that is received for each of the patients in the practice records. Issues related to patient privacy and confidentiality need to be considered in this step in the procedure of data extraction if the operator is not the usual care-provider of the patient. Additionally, while PCS CAT can be easily integrated with all major practice desktop software systems, it has technical compatibility issues with *Profile*, a practice management software used by some practices hence making them ineligible to participate.

## Conclusions

This pilot project has demonstrated that systematic data that is routinely entered into desktop software in General Practice could form the basis of a valid and sensitive surveillance system on chronic diseases, provided that sufficiently representative samples of sentinel sites are recruited within Medicare Local regions.

## Abbreviations

DoHA: Department of Health and Ageing; GP: General Practice; ISML: Illawarra Shoalhaven Medicare Local; SCFH: Shell Cove Family Health; LHDs: Local Health Districts; ABS: Australian Bureau of Statistics; LGAs: Local Government Areas; SLAs: Statistical Local Areas; MLs: Medicare Locals; NHS: National Health Surveys; ISLHD: Illawarra Shoalhaven Local Health District; PHIDU: Public Health Information Development Unit; NSW: New South Wales; PCS CAT: Pen Computer Systems Clinical Audit Tool; SNOMED-CT: Systematized Nomenclature of Medicine Clinical Terms; DOCLE: Doctor Command Language; ICPC2+: International Classification for Primary Care 2+; RACGP: Royal Australian College of General Practitioners; AHS: Australian Health Survey; MBS: Medicare Benefit Scheme; NPI: National Performance Indicator; NHANES: National Health and Nutrition Examination Surveys; CDC: Centers for Disease Control and Prevention; AUSDRISK: Australian Type 2 Diabetes Risk Assessment Tool.

## Competing interests

The authors declare that they have no competing interests.

## Authors’ contributions

AG formulated the study design and conceptualization, performed data extraction and statistical analysis and drafted the paper. KEC contributed to study design and conceptualization, and provided editorial input and data interpretation, and conducted a literature review of the topic. LG trained and educated practice staff in undertaking the data cleansing of their practice clinical database. MB reviewed the statistical methodology and data analysis, and contributed to editing the final version. KMD enabled the coordinated implementation of the study protocol at the practice and helped to draft the manuscript. All authors read and approved the final manuscript.

## Pre-publication history

The pre-publication history for this paper can be accessed here:

http://www.biomedcentral.com/1471-2296/14/109/prepub

## References

[B1] Department of Health and Ageing (DoHA), GP Super Clinics [http://www.health.gov.au/internet/main/publishing.nsf/Content/pacd-gpsuperclinic-about]

[B2] Shell cove family health[http://www.scfh.org.au/about-us.php]

[B3] Australian Bureau of Statistics (ABS), Health Survey (National) [http://www.abs.gov.au/AUSSTATS/abs@.nsf/DSSbyCollectionid/DA11205FB55BD4F4CA256BD000272190?opendocument]

[B4] NSW Ministry of Health, New South Wales Population Health Survey [http://www.health.nsw.gov.au/publichealth/surveys/phs.asp]

[B5] Ghosh A McDonald K Marshall K Illawarra-Shoalhaven Medicare Local – Population Health Profile: 2013 2013 NSW: Grand Pacific Health Ltd

[B6] NSW Health, Local Health Districts [http://www.health.nsw.gov.au/lhd/pages/default.aspx]

[B7] Newell S Girgis A Sanson-Fisher RW Savolained NJ The accuracy of self-reported health behaviors and risk factors relating to cancer and cardiovascular disease in the general population: a critical review Am J Prev Med 1999 17 3 211 229 10.1016/S0749-3797(99)00069-0 10987638

[B8] O’Toole B Driver B Britt H Bridges-Webb C Using general practitioners to measure community morbidity Int J Epidemiol 1991 20 1125 1132 10.1093/ije/20.4.1125 1800413

[B9] Pearson N O’Brien J Thomas H Ewings P Gallier L Bussey A Collecting morbidity data in general practice: the Somerset morbidity project BMJ 1996 312 1517 10.1136/bmj.312.7045.1517 8646146 PMC2351269

[B10] Alderson M Mortality, morbidity and health statistics 1988 London: Stockton

[B11] Fleming DM The measurement of morbidity in general practice J Epidemiol Community Health 1991 45 180 183 10.1136/jech.45.3.180 1757757 PMC1060754

[B12] Brage S Bentsen BG Bjerkedal T Nygard JF Tellnes G ICPC as a standard classification in Norway Fam Pract 1996 13 391 396 10.1093/fampra/13.4.391 8872099

[B13] Chauvin P Valleron A Participation of French general practitioners in public health surveillance: a multidisciplinary approach J Epidemiol Community Health 1998 52 suppl 1 2s 8s 9764263

[B14] Milne RM Taylor MW Taylor RJ Audit of populations in general practice: the creation of a national resource for the study of morbidity in Scottish general practice J Epidemiol Community Health 1998 52 Suppl 1 20S 24S 9764266

[B15] de Grauw WJC van den Hoogen HJM van de Lisdonk EH van Gerwen WHEM van Weel C Control group characteristics and study outcomes: empirical data from a study on mortality of patients with type 2 diabetes mellitus in Dutch general practice J Epidemiol Community Health 1998 52 suppl 1 9s 12s 9764264

[B16] The Royal Australian College of General Practitioners Standards for general practices 2010 4 South Melbourne: The RACGP [http://www.racgp.org.au/your-practice/standards/standards4thedition/practice-services/1-7/health-summaries/]

[B17] Australian Bureau of Statistics, Australian Health Survey First results, 2011–12 cat. no. 4364055001 [http://www.abs.gov.au/AUSSTATS/abs@.nsf/Lookup/4364.0.55.001Main+Features12011-12?OpenDocument]

[B18] Australian Bureau of Statistics Population by Age and Sex, regions of Australia 2011 cat. no. 32350 [http://www.abs.gov.au/ausstats/abs@.nsf/mf/3235.0]

[B19] Department of Health and Ageing, MBS Online Medicare benefits schedule 2013 [http://www.mbsonline.gov.au/]

[B20] Walley T Mantgani A The UK general practice research database Lancet 1997 350 1097 1099 10.1016/S0140-6736(97)04248-7 10213569

[B21] Szeles G Voko Z Jenei T Kardos L Pocsai Z Bajtay A Papp E Pasti G Kosa Z Molnar I Lun K Adany R A preliminary evaluation of a health monitoring programme in Hungary Eur J Pub Hlth 2005 15 26 32 10.1093/eurpub/cki107 15788800

[B22] Fleming DM Schellevis FG Paget WJ Health monitoring in sentinel practice networks 2001 European Commission: Final Report. Luxembourg 10.1093/eurpub/13.suppl_1.80 14533754

[B23] Pringle M Hobbs R Large computer databases in general practice BMJ 1991 312 741 742 2021762 10.1136/bmj.302.6779.741 PMC1669516

[B24] Crim MT Yoon SS Ortiz E Wall HK Schober S Gillespie C Sorlie P Keenan N Labarthe D Hong Y National surveillance definitions for hypertension prevalence and control among adults Circ Cardiovasc Qual Outcomes 2012 5 3 343 351 10.1161/CIRCOUTCOMES.111.963439 22550130 PMC3407684

[B25] Journal Officiel de la République Française Loi n° 2004–806 du 9 août 2004 relative à la politique de santé publique Journal Officiel du 2004 Official Gazette No. 185 of 11 August 2004, Text 4, NOR: SANX0300055L

[B26] Metzger MH Durand T Lallich S Salamon R Castets P The use of regional platforms for managing electronic health records for the production of regional public health indicators in France BMC Med Inform Decis Mak 2012 12 28 42 10.1186/1472-6947-12-28 22471902 PMC3378443

[B27] Centers for Disease Control and Prevention Public health surveillance and informatics program[http://www.cdc.gov/osels/phsipo/index.html]

[B28] Friedman D Parrish R The population health record: concepts, definition, design and implementation J Am Med Inform Assoc 2010 17 4 359 366 10.1136/jamia.2009.001578 20595299 PMC2995645

[B29] International Organization for Standardization: ISO/TR 20514 Health informatics - electronic health record - definition, scope, and context 2005 Geneva: ISO

[B30] Department of Health and Ageing (DoHA) Medicare health assessments resource Kit [http://www.health.gov.au/internet/main/publishing.nsf/Content/mha_resource_kit]

[B31] Department of Health and Ageing (DoHA) Prevention of type 2 diabetes program [http://www.health.gov.au/internet/main/publishing.nsf/Content/chronic-diab-prev-aus]

[B32] ANPHA Australian national preventive health agency strategic plan 2011 – 2015 2011 Canberra: Commonwealth of Australia

[B33] Khan NF Harrison SE Rose PW Validity of diagnostic coding within the general practice research database: a systematic review Br J Gen Pract 2010 60 572 e128 e136 10.3399/bjgp10X483562 20202356 PMC2828861

[B34] National E-Health Transition Authority Practice incentives program (PIP) eHealth incentive - 1 February 2013 compliance checklist [http://www.racgp.org.au/download/Documents/e-health/201212epipeligibilitychecklist.pdf]

